# Role satisfaction among community volunteers working in mass COVID-19 vaccination clinics, Waterloo Region, Canada

**DOI:** 10.1186/s12889-023-15597-9

**Published:** 2023-06-21

**Authors:** Moses Tetui, Ryan Tennant, Alexander Patten, Ben Giilck, Catherine M Burns, Nancy Waite, Kelly Grindrod

**Affiliations:** 1 School of Pharmacy, University of Waterloo, Kitchener, ON Canada; 2 Department of Epidemiology and Global Health, Umeå University, Umeå, Sweden; 3 Systems Design Engineering, Faculty of Engineering, University of Waterloo, Waterloo, ON Canada; 4 Department of Biology, University of Waterloo, Waterloo, ON Canada; 5 Department of Physics and Astronomy, Faculty of Science, University of Waterloo, Waterloo, ON Canada; 6 School of Pharmacy, Faculty of Science, University of Waterloo, Kitchener, ON Canada

**Keywords:** Unpaid community volunteers, COVID-19 mass vaccination, Pandemic, Role satisfaction, Public health, Volunteering in emergencies

## Abstract

**Introduction:**

Unpaid community volunteers are a vital public health resource in times of crisis. In response to the COVID-19 pandemic, community volunteers were mobilized to support mass vaccination efforts in many countries. To have this group’s continued engagement, it is essential to understand the community volunteer experience, including the opportunities and challenges they encounter and how these contribute to their role satisfaction. This qualitative study investigated the factors contributing to community volunteers’ role satisfaction at COVID-19 mass vaccination clinics in the Region of Waterloo, Canada.

**Methods:**

Qualitative data were analyzed from 20 volunteers (aged 48–79 years) who had worked at one of four COVID-19 vaccination clinics in the Region of Waterloo, Canada. Data were analyzed thematically using an inductive coding process followed by an iterative process of grouping and identifying linkages and relationships within the themes.

**Results:**

Four interrelated themes were developed from the inductive analysis process. The theme of community volunteers feeling valued or disesteemed in their role depends on the interaction between the three themes of role description, role preparation, and clinic context.

**Conclusions:**

For volunteers in crises such as the COVID-19 pandemic, volunteer role satisfaction depends on how their contributions are valued, the clarity of their role descriptions, volunteer-specific training, and the sentiments of volunteers and staff within the clinic context. Greater role satisfaction can help with retention as volunteers become more resilient and adaptable to the complex dynamic circumstances of a crisis response. Activities such as training and materials development for role preparations should be explicitly planned and well-resourced, even in crisis/pandemic situations. Building clinic managers’ or supervisors’ skills in communication during crisis/pandemic situations and the skills for the creation of team cohesion are critical investment areas.

**Supplementary Information:**

The online version contains supplementary material available at 10.1186/s12889-023-15597-9.

## Introduction

Unpaid community volunteers are critical for global pandemic prevention and control [1–8]. Volunteerism refers to non-obligatory unremunerated activities undertaken in an organized context to benefit others or society [3, 7]. The COVID-19 pandemic has prompted human history’s most remarkable mass vaccination effort. Within a year of vaccine approval, nine billion vaccine doses had been administered worldwide [9]. To achieve this, hybrid approaches to vaccination were employed, leveraging community-based health clinics, pharmacies and mass vaccination sites, which enabled the rapid vaccination of populations [10]. Given the scale of the rollout, many sites relied heavily on the efforts of volunteers. The benefits and challenges experienced by community health volunteers around the world are well described in the literature [5, 11, 12].

The Canadian volunteer sector is substantial, with 79% of individuals aged 15 and older reporting having volunteered (formally or informally) in 2018 [13]. On average, volunteers dedicated 111 h per year to hospitals and 58 h per year to health organizations, demonstrating the significant role of volunteers in public health and health service delivery in Canada [13, 14]. In Canada, health sector volunteers contribute to initiatives related to communicable and non-communicable diseases, health promotion, palliative care, and emergency response programs, among others [15–17]. Volunteers across Canadian provinces provide emergency response and emotional, social, practical, spiritual, and grief support to clients in homes and hospitals [15, 17]. Beyond healthcare, many Canadians also volunteer in social services, recreation, arts, culture, and religion, including virtual volunteer options that emerged during the pandemic [3, 14, 18]. In many examples, volunteer burnout and turnover limit continuity and impact [16].

While volunteers’ experiences are well described globally, there is little research on the factors that influence the role satisfaction of volunteers during public health emergencies like a pandemic [5, 19, 20]. Early research with volunteers suggests that prosocial behaviour may contribute to psychological well-being and role satisfaction in short-lived public health emergencies [5, 21]. However, more information is needed to inform ongoing efforts, given the protracted nature of the COVID-19 pandemic. Additionally, community volunteers in non-pandemic times are often drawn from the same community pool as during the pandemic. Ensuring they are motivated to continue volunteering is essential for both pandemic and non-pandemic efforts. This study qualitatively examined the factors contributing to community volunteers’ role satisfaction at COVID-19 mass vaccination clinics.

## Methods

### Study design, selection of interviewees, and data collection technique

This qualitative study explored the motivations, roles, and experiences of community volunteers working at mass COVID-19 vaccination clinics in the Waterloo Region, Canada. The participants worked in three large and one small COVID-19 vaccination clinic in the Waterloo Region. The Waterloo Region is centrally located in southwestern Ontario, Canada and comprises three cities—Kitchener, Waterloo, and Cambridge—and four townships—North Dumfries, Wellesley, Wilmot, and Woolwich. As of 2020, the region had over 620,000 residents, making it one of the most populated in Canada [22].

The volunteers selected to participate in the study were randomly drawn from volunteers who expressed interest in participating in the study via email. Our selection ensured the maximum variation principle of qualitative research by selecting volunteers across three mass clinics [23]. Twenty volunteers were individually interviewed (in English for 45–60 min) via the Microsoft Teams platform. By this number, and using a semi-structured interview guide, saturation had been reached. In addition, demographic characteristics, including age, gender and employment status, were collected. All interviews were audio recorded and later transcribed in preparation for analysis.

### Data analysis

The data were analyzed thematically. After every 2 to 3 interviews, MT and RT reflected on the interviews and the memos they took while interviewing. This approach supported the decision to end the data collection at a point when both MT and RT noted saturation [24–26]. The cleaned transcripts were exported to MAXQDA (VERBI GmbH, Version 2020.4.1) for open coding.

The inductive open coding process was undertaken collaboratively by three authors (RT, AP, and BG) and guided by MT, a more experienced qualitative researcher. Initially, the authors met to develop a shared understanding of open coding. Open coding was applied to summarize the data line by line with minimal abstraction. The open codes thus were as close as possible to the actual text and descriptive enough to allow basic conceptualization of the original text. To start the open coding, one of the transcripts was independently coded by four authors (MT, RT, AP, and BG); then, a discussion occurred to compare findings and create a common codebook. This collaboratively rich iterative discussion involved dropping, renaming, and merging some codes. The final codebook had a total of 162 open codes. RT, AP, and BG divided the remaining interviews for separate coding. Any new codes would be posted and added to the codebook when identified. Before code groupings were produced, approximately 350 open codes were added to the shared codebook. In addition, the coding process was supported through a weekly reflection meeting led by MT; in this meeting, challenges and new codes were discussed, which gave the whole analysis team a collective understanding of the open coding process.

Completed open codes were exported into an Excel spreadsheet, grouped, and regrouped independently by MT, RT, AP, and BG. Codes that were similar or had linkages were grouped, discussed, and labelled. This led to the creation of 30 labels, categorized into nine sub-themes and four themes (Table 1), which were shared with senior team members KG, CB, and NW for further guidance. The discussion yielded an agreement on the themes and sub-themes names, meanings, and inter-relationships, concluding the analysis process.

**Table 1:** List of labels, sub-themes and themes

Labels	Sub-themes	Themes	
Appreciated, supported, rewarded	*Valued*		**Role satisfaction**
Not valued, undervalued, overwhelmed, lack of support	*Disesteemed*		
Responding to client queries, providing comfort to clients, monitoring clients for adverse reactions	*Psychological support to clients*		**Role description**
Establishing a positive clinic experience, managing clinic flow, cleaning	*Support for clinic logistics*		
Past experiences, selfish reasons, safe activity, making a contribution	*Reasons for volunteering*		**Role preparation**
Self-directed learning, overwhelming learning material, topics covered,	*Asynchronous learning*		
On-the-job learning, learning by doing, uncertain learning environment, flexibility required, keeping up to date, limited preparation	*Adaptative learning*		
Positive communication aspects, negative communication aspects	*Situational awareness*		**Clinic context**
Working as a team, team conflict and tension	*Teamwork*		

## Results

**Fig. 1:**
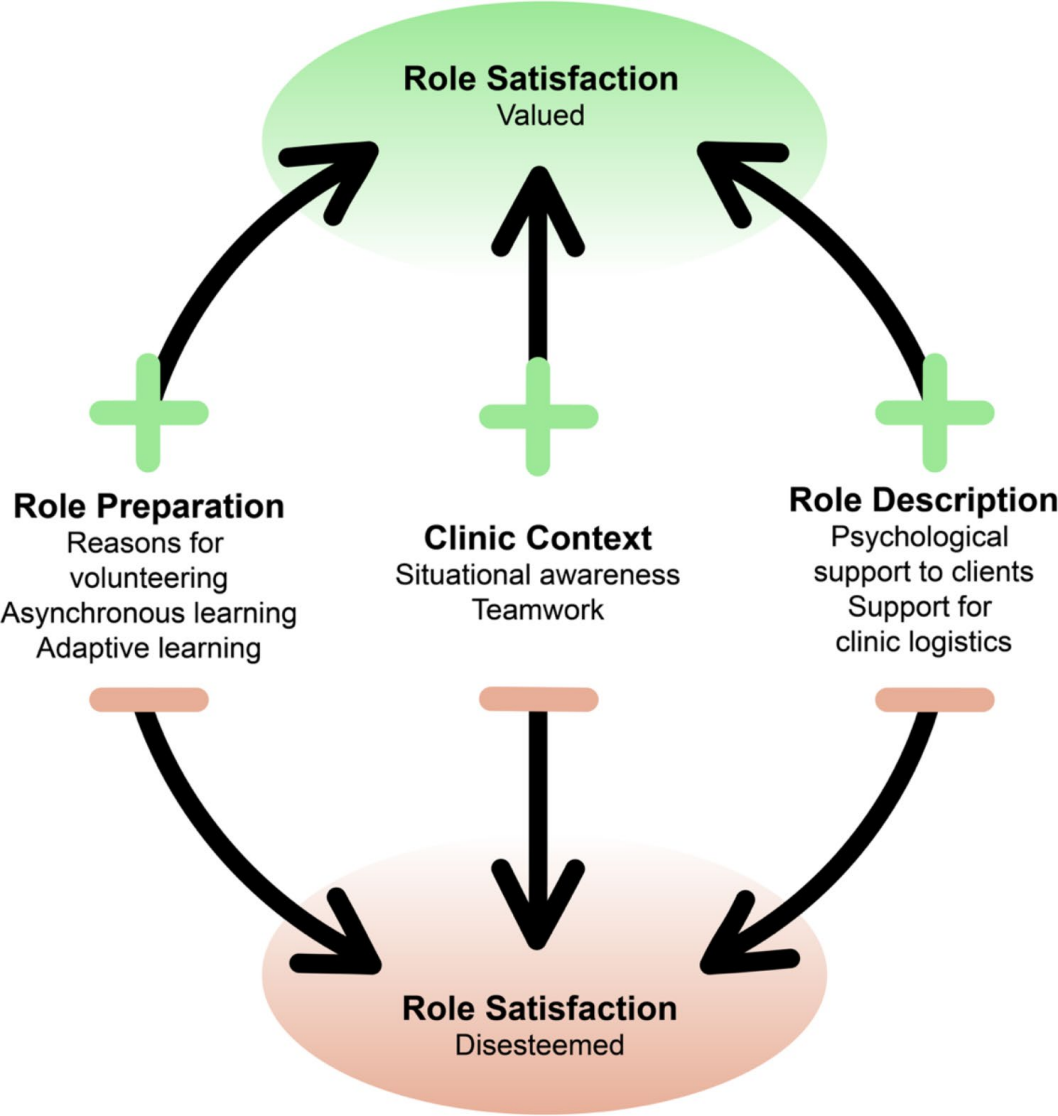
Interrelated themes exploring role satisfaction among community volunteers

The interviewees’ mean age was 64 years (range 48–79 years). Twelve identified as female, while eight identified as male. Most interviewees were retired, with four employed. Four interrelated themes were developed after the inductive analysis (Fig. 1). Role satisfaction was influenced by the way volunteers experienced role description and role preparation. The clinic context described volunteers’ situational awareness (communication) within the clinics and teamwork, and this further reinforced or detracted from their degree of role satisfaction (Fig. 1).

### Role satisfaction

The role satisfaction depicts volunteers’ experience working in the COVID-19 vaccination clinics. The theme is a product of the interaction between role description and role preparation within a specific clinic context, consisting of two sub-themes: valued and disesteemed.

***Valued*** was experienced by the volunteers as being appreciated, rewarded, and supported by the clinic staff and clients. Being asked for input into the daily running of the clinic and being thanked by clinic staff, clients and supervisors made volunteers feel positively seen.

*“I think we were always listened to, and everything was appreciated… All the supervisors are really nice people. Absolutely feel appreciated, like there’s no question and even the staff, they see us as part of the whole solution.” (V06).*

Similarly, making a difference during a challenging pandemic was rewarding. Volunteers were happy to support the COVID-19 vaccination clinics as it provided a sense of fulfillment:

*“I felt good about my decision to volunteer… It was just unexpected appreciation and like I was a small part of some important event. It felt very worthwhile… It was a really positive experience.” (V20).*

Receiving assistance from clinic staff and clients made volunteers feel supported. Clients’ respect for each other also supported the volunteering role, specifically when they accommodated those with accessibility needs. Volunteers felt supported when staff were accessible and approachable for assistance, if staff checked in on them while working, and if staff were responsive to dire situations:

*“There was a lady… She was really angry… Maybe her being anxious showed as anger. Sometimes we get people like that, but it was the first time someone yelled in my face. At that moment the supervisor stepped in and saved the situation. That really felt good.” (V05).*

***Disesteemed*** conversely meant volunteers felt disrespected, overwhelmed, and lacked support from the clinic staff. The volunteer participants felt disrespected when supervisors treated them differently than the staff. For example, when staff were wearing cloth vs. medical masks, when volunteers perceived they were working harder than paid employees, and when volunteers were not consulted before changing their roles.

*“We get emails all the time: ‘Please sign up for more shifts!’ But when it comes to any changes that affect us, nobody sends an email out saying, ‘We’re thinking of moving to four shifts. Do you have any comments about it?’” (V03).*

The volunteers also experienced overwhelming situations impacting role satisfaction. For example, vaccinating teenagers [12–17] was more emotionally draining as it required greater psychological support. Exhaustion was often exacerbated when the clinics were jam-packed, when staff took breaks, or when volunteers missed their shifts.

*“Whenever I get asked to work an extra shift… it seems that there aren’t enough volunteers. I’m totally exhausted.” (V04).*

Lastly, some volunteers found it hard to contact supervisors when they needed help. Shifts were often overlapping, and supervisors differed for each shift in some clinics, making it difficult to find specific supervisors and time to interact with them. This worsened their frustration with the lack of support and made it difficult to feel they belonged:

*“So yeah, [clinic supervisors] don’t have that free time. There’s been one or two issues where I’ve talked to them and said, ‘I’ll just wait out by the security guards, and we’ll just talk outside later,’ but they never come.” (V07).*

### Role description

Volunteers were primarily tasked with psychological support to clients and providing support for client logistics. As indicated earlier, the clarity of these roles and the level of preparedness the volunteers felt impacted their role satisfaction either positively or negatively, as shown in Fig. 1.

***Psychological support to clients*** presented a critical balancing act. Volunteers comforted concerned or anxious clients without overstepping their knowledge and responsibilities. Volunteer responsibilities were primarily client-focused, and a comfortable client experience was an important goal, especially since volunteers were their first contact point. Additionally, volunteers monitored clients after their vaccination. Therefore, responding appropriately to the various emotions or questions expressed by clients was a challenge faced by the volunteers. The extent to which they felt competent to undertake this role, therefore, impacted their role satisfaction.

*“If I had a question that I didn’t know the answer to… I would go to the lead that was on shift that day and ask them. And often the doctors would come… if you had medical questions.” (V18).*

Many volunteers emphasized the importance of empathy in their roles, especially given the large variability in the personality or temperament of clients and their attitudes toward vaccines and their implementation, which were not necessarily positive among all who sought vaccination. The volunteers spoke highly of clients; however, some clients directed outbursts of anger toward volunteers, often about wait times. Clients were also described as judgemental or biased and reported discrimination against volunteers. Needle anxiety and generalized anxieties concerning vaccines or the pandemic were more commonly encountered than uncooperative or ‘difficult’ individuals, where volunteers felt responsible for detecting anxieties and addressing them. Successfully supporting clients in these situations was very rewarding for the volunteers, increasing their role satisfaction.

*“As volunteers, we chat with these young people and try to divert them from thinking about what’s happening. I think that’s a very important role…” (V02).*

***Support for clinic logistics*** referred to facilitating client flow, often described as ‘people-moving.’ The volunteers described their responsibility for establishing a positive clinic experience, a seamless clinic flow, and maintaining clinic cleanliness.

A positive clinic experience was established by welcoming clients upon entry, screening them for COVID-19 symptoms, swapping their masks for medical ones, and confirming their appointment. After vaccination, this experience continued by showing clients how to record their vaccine receipts on their mobile devices and reminding them about their next appointment, if needed. Volunteers explained the steps of the clinic process to prevent confusion and ensure clients would be confident throughout the clinic; this facilitated efficient flow and reduced anxiety. Though with a subtle effect, simple reminders, like telling clients to have their health cards ready, significantly influenced clinic efficiency and client experience, reinforcing volunteer role satisfaction.

*“So, you might be greeting people… or moving people from registration into a vaccination booth. Or getting them to the recovery and… giving people the information they need to know, that’s how I made my positive contribution.” (V12).*

Regarding clinic flow, volunteers managed queues and guided clients through the clinic, which could be challenging. Clients were often confused, and some were uncooperative. Conflicts over line order occasionally emerged and sometimes required a third party, such as a supervisor, to resolve.

*“I called one person and another person started coming up and I waved her back, and then she got very offended… I said, ‘I’m sorry for the confusion, but we’re doing our best.” (V07).*

Finally, volunteers also had cleaning duties. Recommendations concerning sanitation and what constitutes a sufficiently disinfected surface evolved; volunteers needed to adapt to changes that were often abrupt and sometimes poorly communicated and differed between clinics, which sometimes negatively impacted their role satisfaction.

*“At Clinic A, the person checking them out would just wipe [surfaces] down with a wipe. While at Clinic B, a volunteer would come running in and wipe down the whole surface and then discard the wipe.” (V13).*

Despite their generally positive role satisfaction, the participants felt they could have offered more, given their skills and experience.

*“…a lot of volunteers have a lot of different backgrounds that could maybe help a bit more, and we would be more than willing to do it.” (V06).*

Other interviewees mentioned their experience coordinating volunteer programs, working in emergencies, and organizational design. They felt they could have handled more than they were asked to, impacting their engagement and lowering their role satisfaction. Nonetheless, some volunteers found ways to contribute more to their clinics.

*“I’ve got a background in event organization stuff. I have also helped with reinventing the lineup here so that things run more smoothly. There are those cross-talents that have come up along the way – around the languages as well. At [Clinic A], there’s a list every day of the languages that people speak, including sign language, and so that was sort of a natural percolation to be able to help and assist as well.” (V09).*

### Role preparation

This theme includes reasons for volunteering, asynchronous learning, and adaptive learning. This theme spoke to how volunteers were prepared for the roles described in the role description theme above and how this reinforced their positive or negative role satisfaction.

***Reasons for volunteering*** included volunteers’ motivations to participate in the COVID-19 vaccination clinics and how that affected their overall role satisfaction. The participants provided a range of reasons for starting to volunteer; having a positive prior experience as a volunteer in a different context, contributing to the fight against the pandemic, getting vaccinated sooner, and interacting with others. They also felt that volunteering at a clinic was safer—given the rigorous safety protocols—than visiting family or friends whose behaviours one could not control. In addition, some volunteers were simply motivated because they were retired and enjoyed working with people in the community.

*“I didn’t know how to make a contribution [to ending the pandemic] and when I saw the opportunity, I thought this is something I can do.” (V10).*

The reasons for which people volunteered generally influenced their role satisfaction. For example, prior volunteer experience gave some participants the necessary skills to manage what others might consider ‘unpleasant experiences,’ which generally increased their role satisfaction.

*“I have been volunteering at the hospital for many years, I really like to volunteer, so that was something that I would say helped me manage some of the difficult situations compared to other.” (V08).*

On the other hand, some volunteers were initially interested in volunteering because of a self-serving motivation to get ahead of the vaccination queue. Such volunteers easily grew frustrated with the uncertainty that surrounded clinic operations and limited role preparation that was provided.

*“You know, like that’s a bit annoying [double checking of clients’ health cards], but I later understood the reason for asking that and I would try to do that. The clients were frustrated, so maybe they would kind of help put some context to it so that client would be less irritable.” (V20)*.

***Asynchronous learning*** involved sharing training materials with volunteers, allowing them to learn task-specific guidelines and skills within specified timeframes. Learning was self-driven. Topics included using gender-sensitive language, de-escalating a tense situation, communicating vaccine-related messages, monitoring for adverse vaccine reactions, and creating a positive clinic atmosphere. However, in some clinics, learning materials were not role specific but sent to all clinic staff and volunteers—such materials were less prioritized by volunteers. Overall, the volunteers found the training materials were sufficient, while others felt overwhelmed by many reading materials. Therefore, how much one engaged with the materials varied across the volunteers and influenced their role satisfaction accordingly.

*“It was a lot of work, you know, everything from how to deal with people to sexual harassment. So, there was a fair amount of work, extensive amount of training, it was almost too much, I’m retired now, but I feel it was a lot for my role as a volunteer.” (V06).*

***Adaptive learning*** was what volunteer participants referred to as ‘on-the-job training.’ According to interviewees, the COVID-19 vaccination clinic protocols were uncertain and dynamic. The changing vaccination clinic situation meant there were no clear job descriptions, and volunteers had to keep adapting their learning to current conditions. Also, every volunteer shift had different supervisors, and clinic staff shared clinic adaptations with the volunteers at shift debriefs, resulting in different knowledge being shared.

*“I don’t know whether it is the same elsewhere. There are really no job descriptions, so what you know depends on who trains you. If I am trained by a crappy person, then I will be more crappy myself.” (V03).*

Additionally, some vaccine rollout changes made at provincial or federal levels became known to the volunteers, clinic staff, and clients simultaneously. Therefore, learning on the go was a joint undertaking and required volunteers to be flexible and agile to perform their roles successfully. For example, when a policy was made to allow clients to have a mix of the mRNA vaccine brands for dose one and dose two, volunteers recounted how they had to quickly learn to manage angry clients who preferred one vaccine brand over another.

*“I appreciated that the clinic lead would say, ‘We’ve never done this before, so you need to be flexible with us, and we’ll be flexible with each other.’ We had to just learn on the job” (V15).*

Lastly, the interviewees noted that they did not receive sufficient preparation for some of their roles, such as monitoring clients for adverse effects and how to handle some vaccine-related questions. Referring the clients to staff did not always work well because sometimes the appropriate clinic staff would not be readily available, and it presented the volunteers as poorly prepared. This created client frustration and a loss of trust in the volunteers’ level of competence, respectively, thereby negatively affecting their role satisfaction.

*“Some people learn by tacit experiences; people have different learning abilities. So, assuming people will be thinking on the spot without any guidance was not good. I don’t think there’s anything specific on an individual case, like maybe how to deal with an emergency in case help is not nearby. Or maybe dealing with an anxious person, I just felt not prepared enough.” (V01).*

### Clinic context

The context (situational awareness and teamwork) of volunteering in a vaccination clinic influenced the volunteers’ experiences and their role satisfaction, either positively or negatively, as indicated in Fig. 1.

***Situational awareness*** was influenced by the positive and negative aspects of communication within a clinic. When volunteers were made aware of clinic adaptations through daily debriefs, this contributed to a positive experience, enabling them to perform their roles more competently, increasing their motivation and overall role satisfaction.

*“[The staff] do an extraordinary job of communicating with us. For example, they really did a good job of helping us get some language around if somebody had Moderna first and now it was going to be Pfizer, and they had some initial concerns.” (V15).*

Smaller clinics enabled more meaningful communication between volunteers and clients. Chatting with clients was viewed as improving their experience while simultaneously being enjoyable for volunteers.

*“We’re a small town, and we’re not shoving people through like a piece of meat. We’re having a little conversation because they might have to wait a minute or two to go inside.” (V17).*

Volunteers expressed positivity toward information sharing from the clinic staff, but they also observed good communication within the clinic. When the volunteers noticed how effectively the staff handled the uncertainties of client intake by maintaining shared awareness, they felt that the clinic was operating successfully, contributing to positive role satisfaction.

*“The clinic staff kept in constant communication to make sure the pharmacy team was drawing up the correct number of doses to match the number of patients coming through, so that all seemed pretty flawless to me.” (V16).*

However, the volunteers also experienced negative aspects of communication that contributed to poor situational awareness and ultimately fueled negative role satisfaction. Miscommunications occurred about responsibilities, expectations, updated protocols, and changes to the clinic workflow, making them feel siloed from other staff’s information.

*“It’s very much ‘we’ and ‘they.’ You have a supervisor of the medical staff, and you have a supervisor of the volunteers. Then something changes, and either we know about it, and the paid staff don’t, or the paid staff have changed something, and the volunteers know nothing about it.” (V03).*

Volunteer participants desired better communication. However, miscommunications could still occur about their tasks, leading to conflicts between staff and other volunteers and requiring supervisor escalation. Confusion about cleaning expectations was one example, where participants explained their experiences navigating updated protocols, especially when aspects of the clinic changed quickly:

*“The last time I was working [I was told] we’re not supposed to dry, and I said, well, actually, I just asked this morning… and was told that no, we are drying.” (V02).*

Clarifying the cleaning policy may have addressed the conflict for one instance. However, this negatively influenced role satisfaction. While receiving new information became challenging in some clinics, volunteers also expressed having fewer opportunities to share feedback or suggestions, and some volunteers wondered if they should start seeking volunteer opportunities elsewhere.

*“And it got to the point where I second guess myself if I have a suggestion to make. If I want to discuss something with them, it’s a pretty short discussion now; it’s not a friendly back-and-forth. Maybe it’s time for me to move on to something else. I just get a feeling it’s not quite the warm fuzzy place it was at one time.” (V19).*

***Teamwork*** meant volunteers had a personal and positive experience working with clinic staff, including interacting with staff to resolve issues. The participants described how their own actions enabled them to break down the invisible barriers that separated the clinic staff from the volunteers. Interacting with other staff on a first-name basis was perceived as having improved the volunteers’ sense of belonging to the clinic team and their role satisfaction.

*“Being more of a people person, I know more of the staff because I talked to them, whereas a lot of volunteers don’t ever talk to the staff, so there’s very much that separation… A lot of the staff know me by name now… I call it the cheers effect.” (V03).*

These relationships sometimes advanced further into collaborative strategy development for improving client flow in the face of changes to the context in which people were arriving for their vaccine:

*“For both first dose and second dose priority lines, I’d huddle with the security and the other volunteers, and we’d say, what are we gonna do? How are we gonna handle this?” (V13).*

Volunteers felt that their roles, while ‘not changing the world,’ were essential to the overall system. Including volunteers in supporting clinic flow improved their sense of belonging and role satisfaction, further supported by clinic leadership.

*“We’re led by a great example. They’re some of the funniest people that I’ve ever met. They got ice cream for each other last week like they’re a good, supportive team. The public-facing relationships that I see are people who really care about each other.” (V10).*

However, sometimes conflicts and tensions among volunteers and staff because of poor direction, left volunteers feeling disconnected and on their own when facing challenging situations. Some volunteers were heard yelling at others or told by other volunteers to stop asking clients for their preferences because of perceived disruptions to client flow. This may have developed because of volunteers’ perceptions of controlling clinic logistics on the fly:

*“This is the good part about volunteers. If we really felt like, within reason, we could do it better, we just did. We didn’t ask for permission. There was a bit of, you know, what are they going to do? Fire us?” (V06).*

Finally, while teamwork tensions arose among volunteers, they were also present between volunteers and the paid clinic staff. Conflicts were exacerbated when issues surrounded the client’s experience and safety. Instead of working together to improve the client experience, the volunteers’ sometimes felt their positive actions were unnecessarily overruled, negatively impacting their role satisfaction.

*“It goes back to the user experience… one young individual, severely autistic, asked me for a private room, and the doctor said, ‘no, no, I got them in the big room.’ Well, that disrupted everybody for 10 minutes when in fact, they should have just not been in the big room. That’s a doctor thing: ‘I got this.’” (V13).*

## Discussion

Volunteer role satisfaction in the COVID-19 vaccination clinics studied was heavily influenced by the clarity of role descriptions, how well volunteers were prepared for their roles and the level of situational awareness and teamwork in clinics. In the dynamic nature of a pandemic or similar crisis, volunteers are critical for supporting the public and the crisis response staff. Role satisfaction may help with volunteer retention and a willingness to volunteer again. It is, therefore, critical to value volunteer contributions by integrating them with paid staff, providing ample role preparation, and supporting volunteers to adapt to changing circumstances and remain resilient over time.

Another Canadian study also found that volunteers want organizations to acknowledge the value of their contributions [20]. This underscores the importance of simple acts of saying thank you and or providing tokens of appreciation, such as a volunteer appreciation meal or gift. Similarly, a study of community health volunteers in rural Uganda emphasized the impact of community appreciation on volunteer role satisfaction [19].

In contrast, a heavy workload, high-performance expectations, and a lack of respect from the community and superiors can hurt role satisfaction, as seen elsewhere [27, 28]. Participants experienced angry outbursts and discrimination from clients, which was a negative aspect of volunteer-client interactions. COVID-19 vaccination clinic volunteers in two other Canadian studies reported experiencing harassment, bullying, and verbal abuse from upset clients [29, 30].

Volunteer Canada suggests supporting volunteers’ mental health and well-being is critical to volunteer organizations navigating the COVID-19 pandemic [20]. Practically speaking, ensuring support from paid staff is readily available, creating an atmosphere of belonging for volunteers, and strict enforcement of policies to prevent workplace harassment and violence are essential to improve and protect volunteer role satisfaction.

Findings from Sinclair et al. (2022) suggest that well-defined roles are more rewarding for volunteers than ambiguous ones [8]. Our study found that pandemic volunteers often had to work with vague role descriptions and rapidly adapt when roles or clinic logistics changed, often with little guidance. Given the unpredictability of COVID-19 and its related policies and the workload of paid staff, strict role descriptions were not always possible [20]. However, our research suggests that clear and regular communication about role descriptions, even in crisis response, may help lessen the burden of volunteer burnout.

Volunteers had intrinsic and altruistic motivations for volunteering during the pandemic, including a sense of moral duty and a desire to help others and give back to society [5, 12]. Assisting volunteers in fulfilling their goals may enhance role satisfaction [11]. However, volunteers were also motivated by personal benefits, such as earlier access to a vaccine or ‘getting out of the house’ and interacting with others, given the isolating stay-at-home and physical distancing orders. Therefore, paying attention to volunteer motivations by intentionally seeking to meet them helps keep role satisfaction levels high.

To prepare for their role, volunteers benefit more from concise, standardized, tailored materials specific to volunteer roles while still providing clear verbal guidance on-the-job. Inadequate communication and training make it difficult for volunteers to prepare for their roles and to adapt to changing pandemic policies [31]. A study by Afulani et al. (2021) found that low perceived preparedness was associated with lower role satisfaction among pandemic healthcare workers, as we found among volunteers [32, 33]. Nonetheless, adaptive learning in an emergency context, such as the COVID-19 pandemic, is critical [31, 34]. Indeed, amidst the pandemic Canadian volunteer organizations struggled to find time to recruit and train volunteers while continuing to provide services [20]. Equipping volunteers with skills to adapt to unstable environments while creating stability through harmonized communication and standardized role preparation processes becomes indispensable [12, 35].

In our study, situational awareness and teamwork issues negatively impacted volunteers’ interest in working in the clinic. Other studies have noted that during the COVID-19 pandemic, cooperation in healthcare simultaneously became more critical and challenging [36]. Team-level stressors, including a lack of familiarity with team members and care for each other, contribute to reduced collective efficacy, vigilance, and resilience, resulting in poor teamwork and suboptimal quality of care [36].

According to the Integrated Resilience Attributes Framework, situated resilience refers to adapting to unexpected events at a micro-level, such as clinic flows and technological difficulties. In contrast, structural resilience refers to reviewing resources and practices to better support work activities at a meso-level [31]. Team-level strategies to build situational and structural resilience include providing peer support and management support and information, communication, and training [31]. In our study, communication significantly contributed to situational awareness, teamwork, and conflict resolution in a clinic context. This complements findings from Zaghini et al. (2021), who report that interpersonal conflicts with colleagues and superiors reduce the role satisfaction of pandemic healthcare workers [37]. Consistent and positive communication with clinic volunteers may increase role satisfaction [12, 35].

## Conclusions and recommendations

This study highlights factors contributing to community volunteers’ role satisfaction at COVID-19 mass vaccination clinics. Role satisfaction is a significant outcome, as the continued commitment of unpaid community volunteers is necessary to sustain long-term vaccination and other public health efforts, especially in emergency and resource-limited settings. Volunteers derive satisfaction from feeling valued, appreciated, and supported by paid staff and clients. Further, volunteers may be more satisfied when there is a clear definition of roles and responsibilities, when volunteer roles are matched to their skills and experiences, and when they are supported in challenging situations. Rather than general staff training, volunteer-specific training is crucial to promote self-directed and adaptive learning in volunteers. Similarly, resources allowing, anticipatory preparedness training of an existing pool of reserve public health volunteers could help circumvent some of the challenges of training in an emergency.

The study provides an interrelated framework to support role satisfaction among volunteers working in emergency pandemic situations. Volunteers play a critical role in enabling the health system to cope with pandemic-related stress and strains; therefore, their role satisfaction must be maintained. This would ultimately enable the entire population to overcome pandemics safely and more quickly by allowing the health system to breathe and to invest in the scarce resources where they are most needed.

## Electronic supplementary material

Below is the link to the electronic supplementary material.

Supplementary Material 1

## Data Availability

Interview transcripts are available by request to the corresponding author.
