# Supplementary figures and images for: Role satisfaction among community volunteers working in mass COVID-19 vaccination clinics, Waterloo Region, Canada
Source: BMC Public Health. 2023 Jun 21;23:1199. doi: 10.1186/s12889-023-15597-9 (PMC10283305; doi:10.1186/s12889-023-15597-9)

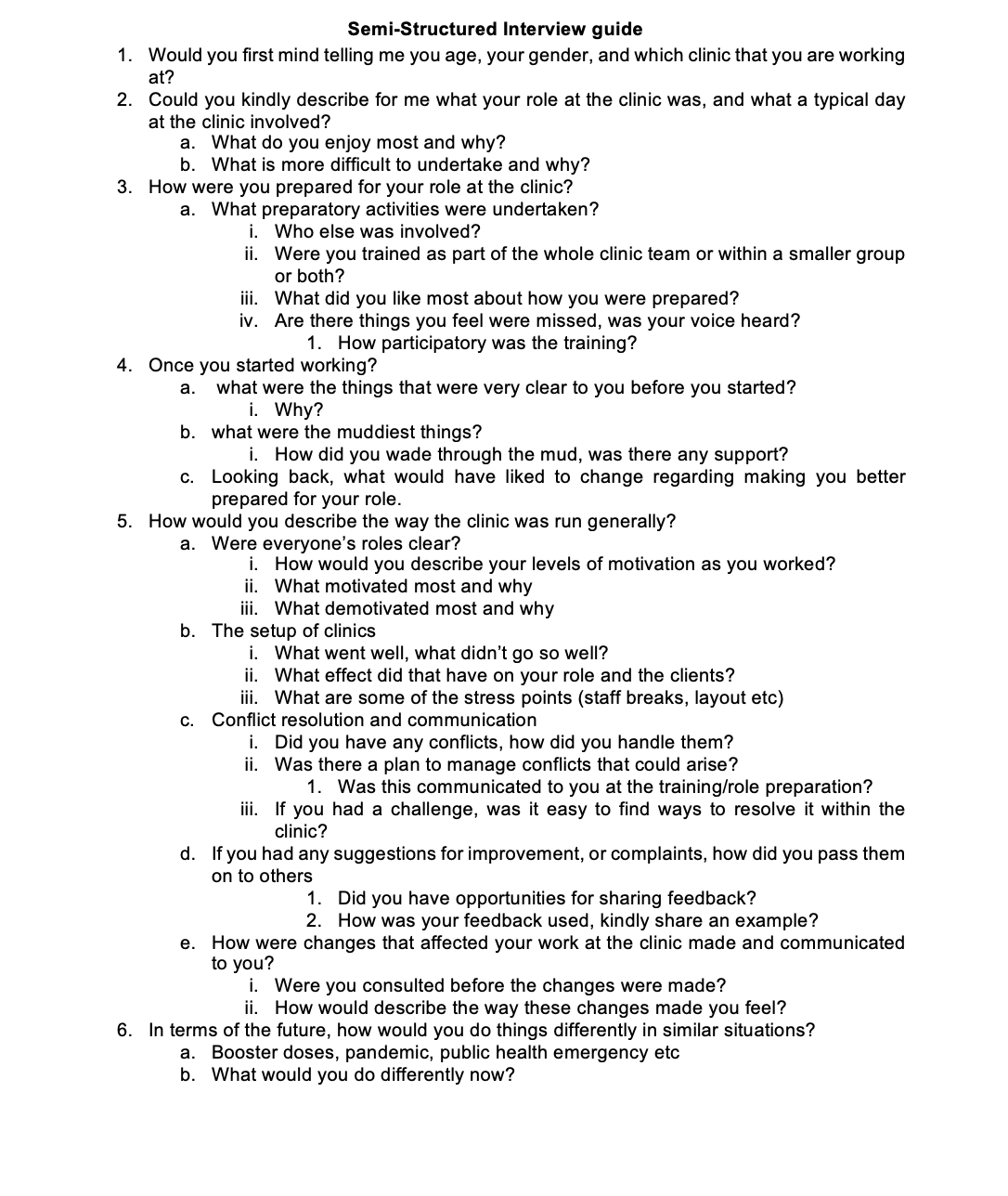

Supplement: Supplementary file 1 — Supplementary Material 1 [file 12889_2023_15597_MOESM1_ESM.png]
